# Identification of Catalytic Residues Using a Novel Feature that Integrates the Microenvironment and Geometrical Location Properties of Residues

**DOI:** 10.1371/journal.pone.0041370

**Published:** 2012-07-19

**Authors:** Lei Han, Yong-Jun Zhang, Jiangning Song, Ming S. Liu, Ziding Zhang

**Affiliations:** 1 State Key Laboratory of Agrobiotechnology, College of Biological Sciences, China Agricultural University, Beijing, People's Republic of China; 2 State Key Laboratory for Biology of Plant Diseases and Insect Pests, Institute of Plant Protection, Chinese Academy of Agricultural Sciences, Beijing, People's Republic of China; 3 National Engineering Laboratory for Industrial Enzymes and Key Laboratory of Systems Microbial Biotechnology, Tianjin Institute of Industrial Biotechnology, Chinese Academy of Sciences, Tianjin, People's Republic of China; 4 Department of Biochemistry and Molecular Biology, Faculty of Medicine, Monash University, Melbourne, Victoria, Australia; 5 CSIRO - Mathematics, Informatics and Statistics, Clayton, Victoria, Australia; Technical University of Denmark, Denmark

## Abstract

Enzymes play a fundamental role in almost all biological processes and identification of catalytic residues is a crucial step for deciphering the biological functions and understanding the underlying catalytic mechanisms. In this work, we developed a novel structural feature called MEDscore to identify catalytic residues, which integrated the microenvironment (ME) and geometrical properties of amino acid residues. Firstly, we converted a residue's ME into a series of spatially neighboring residue pairs, whose likelihood of being located in a catalytic ME was deduced from a benchmark enzyme dataset. We then calculated an ME-based score, termed as MEscore, by summing up the likelihood of all residue pairs. Secondly, we defined a parameter called Dscore to measure the relative distance of a residue to the center of the protein, provided that catalytic residues are typically located in the center of the protein structure. Finally, we defined the MEDscore feature based on an effective nonlinear integration of MEscore and Dscore. When evaluated on a well-prepared benchmark dataset using five-fold cross-validation tests, MEDscore achieved a robust performance in identifying catalytic residues with an AUC1.0 of 0.889. At a ≤10% false positive rate control, MEDscore correctly identified approximately 70% of the catalytic residues. Remarkably, MEDscore achieved a competitive performance compared with the residue conservation score (e.g. CONscore), the most informative singular feature predominantly employed to identify catalytic residues. To the best of our knowledge, MEDscore is the first singular structural feature exhibiting such an advantage. More importantly, we found that MEDscore is complementary with CONscore and a significantly improved performance can be achieved by combining CONscore with MEDscore in a linear manner. As an implementation of this work, MEDscore has been made freely accessible at http://protein.cau.edu.cn/mepi/.

## Introduction

Enzymes play a fundamental role in fulfilling diverse biochemical functions and are essentially required for almost all cellular processes. Although the catalytic mechanisms of certain enzymes have been well characterized [1], it remains a difficult and challenging task to rationalize the sequence-structure-function relationship and unravel the biological functions of the majority of enzymes. Owing to structural genomics efforts [2], [3], a considerable number of protein structures have been determined. Solving the three-dimensional structure of an enzyme can further deepen our understanding of its catalytic mechanism at the atomic level. However, it is still a challenging task to establish the linkage between the given protein structures and their catalytic mechanisms, reflected by the vast number of functionally uncharacterized enzyme structures generated from the structural genomics projects [4]. As catalytic residues are directly involved in catalytic processes, their identification is the first crucial step to characterize the catalytic mechanism and function of an enzyme. Since experimental determination of catalytic residues from large-scale proteome data is a costly and daunting task, computational methods that are capable of identifying catalytic residues from enzyme sequence and/or structure information play an increasingly important role in complementing the experimental efforts and supporting the functional annotation. Apart from providing critical insights into the rules that govern enzymatic catalysis, the identification of catalytic residues also has important applications in the areas of drug design [5], protein engineering, metabolic pathway analysis and synthetic biology [6].

In the past few decades, intensive efforts have been dedicated to identifying catalytic residues in proteins and many features or parameters have been exploited to characterize the properties of catalytic residues. These features can be generally divided into two categories: sequence- and structure-based. Amino acid residues have different propensities to be catalytic residues in nature. For example, it was previously observed that roughly 65% of catalytic residues were charged (H, R, K, E, D), 27% were polar (Q, T, S, N, C, Y, W), and 8% were hydrophobic (G, F, L, M, A, I, P, V) [7]. Therefore, amino acid residue type is probably the simplest but one of the most efficiently used sequence features to identify catalytic residues. In addition, residue conservation, derived from the multiple sequence alignment (MSA) of a query sequence, has also proved to be one of the most powerful singular features in predicting catalytic residues [8]–[13]. The state-of-the-art residue conservation algorithms include the Shannon entropy-based method [14], Jensen-Shannon divergence method [15], Rate4site algorithm [16] and other methods [17]–[20]. More recently, researchers have found that co-evolutionary features could be commonly derived from the neighboring residues surrounding functionally important sites [21] and such information could be utilized to facilitate the identification of catalytic residues [21]–[23].

Given that enzymes perform their biological functions on the basis of specific three-dimensional structures, a variety of simple structural features have been proposed to characterize catalytic residues. For example, it has been shown that catalytic residues prefer to be located in the geometric centers of the protein structures [24]. They also tend to be located in a large cleft on the protein structure surface [7], [25]. Therefore, the distance of the cleft to the center of protein structure can provide quantitative information for identifying catalytic residues [26]. In addition, as most catalytic residues act as either acceptors or donors in the catalytic process, the hydrogen bonds in protein structures can also be used to discriminate catalytic from non-catalytic residues [8], [27]. Other important structural and dynamic properties, such as solvent accessibility [7], [27], flexibility of loop regions [7], [28] and B-factors [7], [29] have also been used as features or descriptors for predicting catalytic residues.

Recently, more complicated structure-based features have been developed to distinguish catalytic from non-catalytic residues. It has been established in protein engineering that mutations of the active site residues usually lead to an increased stability. Therefore, the properties that describe the destabilizing effects of residues were employed to identify catalytic residues [25], [30]. Likewise, since the electrostatic property is important for an enzyme to maintain its function, the electrostatic property-based features derived from the titration curves of residues [31], [32] and the electrostatic energy of residues [33] have proved useful in predicting catalytic residues. Bryliński et al. observed that the regions with significant irregular hydrophobicity in enzyme structures tend to be functionally important and thus developed a novel feature based on the Fuzzy Oil Drop model to predict active sites [34]. Sacquin-Mora et al. proposed a force constant-based feature to quantify the easiness of moving a given residue relative to the rest in a protein based on the fact that catalytic residues are generally more rigid than others. They further employed it as an informative feature to detect catalytic residues [35]. In summary, most of these structural features are developed based on physicochemical properties of amino acid residues which typically require intensive dynamics and/or energy calculations. This has greatly limited their high-throughput applications.

New features based on improved representations of protein structures have been proposed in recent years. For example, a protein structure can be represented as a residue interaction network where each residue is represented as a node and two interacting residues are connected by an edge [36]. A network parameter, i.e. the Closeness centrality (also called Closeness), has been demonstrated to be an informative feature in detecting catalytic residues [37]–[39]. The concept of microenvironment (ME) has been previously proposed to describe a residue's local structural environment [40], extracted from the physical and chemical properties of the residue and its structurally neighboring residues at the residue/atom level. The ME-related features have been widely used to recognize catalytic residues in protein structures [41]–[46].

To further improve the prediction performance, some features have been integrated into different predictors using either statistical (e.g. logistic regression [30], [47] and maximum likelihood models [48]) or machine learning methods (e.g. support vector machines [8], [43], [44], [49], [50] and neural networks [10], [27], [51]). In the past few years, we have witnessed the flourish of such integrative predictors [52], [53]. In summary, statistical methods can yield an improved performance based on an efficient integration of individual (which are largely independent) descriptors to simple and interpretable models. In comparison, although machine learning methods can usually lead to a more competitive performance through the use of much larger feature sets, they have certain disadvantages. For example, they are often criticized for lacking biological interpretation of the trained ‘black box’ models and thereby difficult for biologists to readily deploy and understand the predictions of such models.

In this study, we developed a novel structural feature to identify catalytic residues by integrating the ME and geometrical properties of residues. Firstly, we converted the ME of a residue into a series of spatially neighboring residue pairs, whose propensities to occur in the catalytic ME were deduced from a pre-built enzyme dataset. Then, we proposed a new feature called MEscore to characterize the ME of a residue. To the best of our knowledge, this is the first endeavor to represent the ME of a residue using a group of residue pairs. We then proposed and validated another feature called Dscore that quantifies the centrality of a residue to the whole protein structure. As MEscore and Dscore are largely complementary to each other, we further integrated these two features to a novel feature named MEDscore. Remarkably, MEDscore revealed a competitive performance compared with the residue conservation score. In this work, we describe and discuss the construction of MEscore and MEDscore as well as the overall performance assessment of different features in detail. Specially, the fundamental mechanism by which MEscore and MEDscore are informative for catalytic residue recognition is also discussed.

## Results and Discussion

### Propensities of residues in the microenvironment (ME) surrounding the catalytic residues

**Figure 1:**
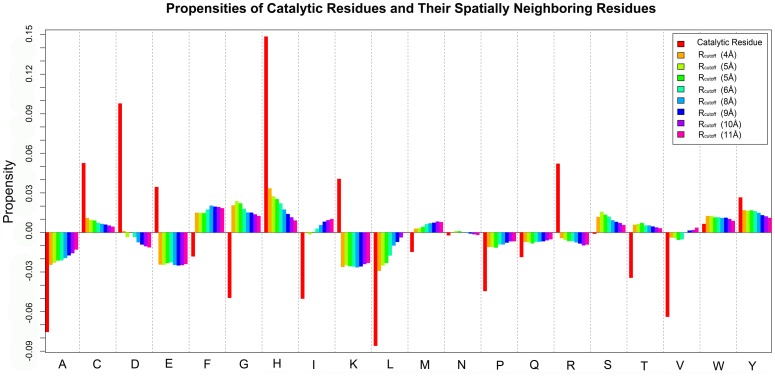
Propensities of 20 amino acids in their roles as catalytic residues and their spatially neighboring residues. The catalytic propensity of any residue is defined as its frequency been a catalytic residue minus its corresponding background frequency. Likewise, the propensity of any residue as catalytic residues' neighbor is defined as its frequency in the MEs of catalytic residues minus the corresponding background frequency. A positive bar means that the residue is enriched, while a negative bar means that the residue is depleted. The distance cutoff (R_cutoff_) values, ranging from 4 to 11 Å at an interval of 1Å, were used to calculate the structural neighbors of catalytic residues. All the residues in the enzyme dataset were used to calculate the background frequency.

We systematically analyzed the amino acid compositions of catalytic residues and their spatially neighboring residues based on a well-prepared enzyme dataset consisting of 223 catalytic domains. More details about this enzyme dataset can be found in the ‘Materials and Methods’ Section. As shown in Figure 1, catalytic residues tend to be either charged or polar residues, which is in accordance with previous studies [7]. Interestingly, we also found that the neighboring residues in the ME surrounding the catalytic residues exhibit remarkably different propensities: some residues (C, M, H, S, T, W, Y, F, G) prefer to be located in the neighborhood of catalytic residues, while others (E, K, R, D, A, L, P, V, Q) are seldom observed to be around catalytic residues. When different distance cutoff (R_cutoff_) values (ranging from 4.0 to 11.0 Å) were applied to assign the structural neighbors of the catalytic residues, the corresponding trends of amino acid compositions in the ME of catalytic residues remain largely unchanged (Figure 1). Such different residue propensities of ME indicate that it is possible to develop a feature that represents ME at the residue level to distinguish catalytic from non-catalytic residues.

If we consider possible biochemical mechanisms and driving forces, the propensities of 20 amino acids in the ME of catalytic residues might reflect the intrinsic requirement for catalytic reactions. For instance, the enrichment of aromatic residues (i.e. F, Y, and W) may be attributed to the fact that their side chains are required to form the cation-π interactions with the charged catalytic residues and/or charged substrates, which is helpful for stabilizing the transition state. Similarly, the enrichment of residue G in the ME of catalytic residues may reduce the steric effects and facilitate the conformational change of catalytic sites, given that conformational changes are needed for substrate binding, protons and/or electrons transport, and product release [54]. We could envisage that the use of these properties will be very helpful for deciphering and understanding ME, which will be discussed in the following sections.

### Statistical analysis of MEscore

Based on the observation that catalytic residues have unique ME features, we further considered converting the ME of a query residue into a series of spatially neighboring residue pairs and proposed a scoring function called MEscore to measure the potential of a query residue being catalytic. To achieve this, we first constructed a 400-dimensional weight coefficient vector, 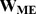, to quantify the likelihood of each residue pair in the ME of catalytic residues, inferred from a pre-built enzyme dataset (the training dataset). To obtain the MEscore for a query residue, we summed over the corresponding coefficients of all residue pairs related to the query residue within a distance threshold. Generally, the higher the MEscore, the higher the probability for the query residue to be catalytic. Details regarding the definitions and calculations of 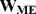 and MEscore can be found in the ‘Materials and Methods’ Section.

**Figure 2:**
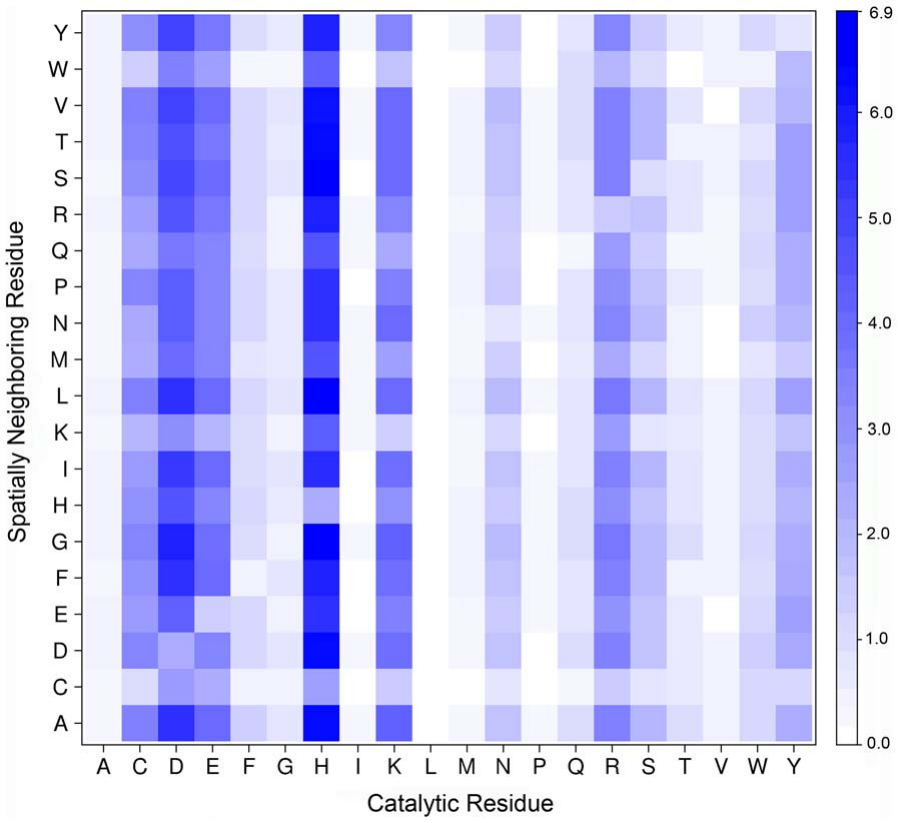
The weight coefficients of spatially neighboring residue pairs in the MEs of catalytic residues. The *x*-axis denotes different catalytic amino acids and the *y*-axis represents the corresponding neighboring residue types occurring in the MEs of catalytic residues. A weight coefficient close to maximum value is color-coded in blue, and it varies continuously to white color as equal to 0.0. Note that the weight coefficients were derived from the whole enzyme dataset (i.e. the 223 enzymes used in this work).

The 400-dimensional weight coefficient vector 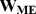, after being converted into a 20×20 matrix, is shown in Figure 2 and Table S1. Different residue pairs exhibit scaled propensities in the ME of catalytic residues, thereby providing important insights into the molecular mechanism of enzymatic catalysis. For instance, the residue D is frequently observed in the ME of catalytic H and R (Figure 2 and Table S1). It has been previously shown that the *pK*a value of the catalytic residue H would increase when there was a structurally neighboring residue D in local structures, as residue D could help the catalytic residue H perform its function as an acid-base [55]. A similar finding is associated with the catalytic residue R. In catalytic processes, the residue R usually plays a stabilizing role [56]. Its spatially neighboring residue D, which has opposite charge to residue R, helps to stabilize charge concentration [55]. Moreover, the salt bridges or hydrogen bonds formed between the catalytic residue R and its spatially neighboring residue D also facilitate the stabilization. In contrast to the amino acid compositions of catalytic residues and their spatially neighboring residues, 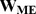 clearly quantifies the preference of a residue pair in the ME of catalytic residues (Figure 2). This implies that the transformation of ME into the combination of residue-residue pairs should be more informative in distinguishing catalytic from non-catalytic residues.

**Figure 3:**
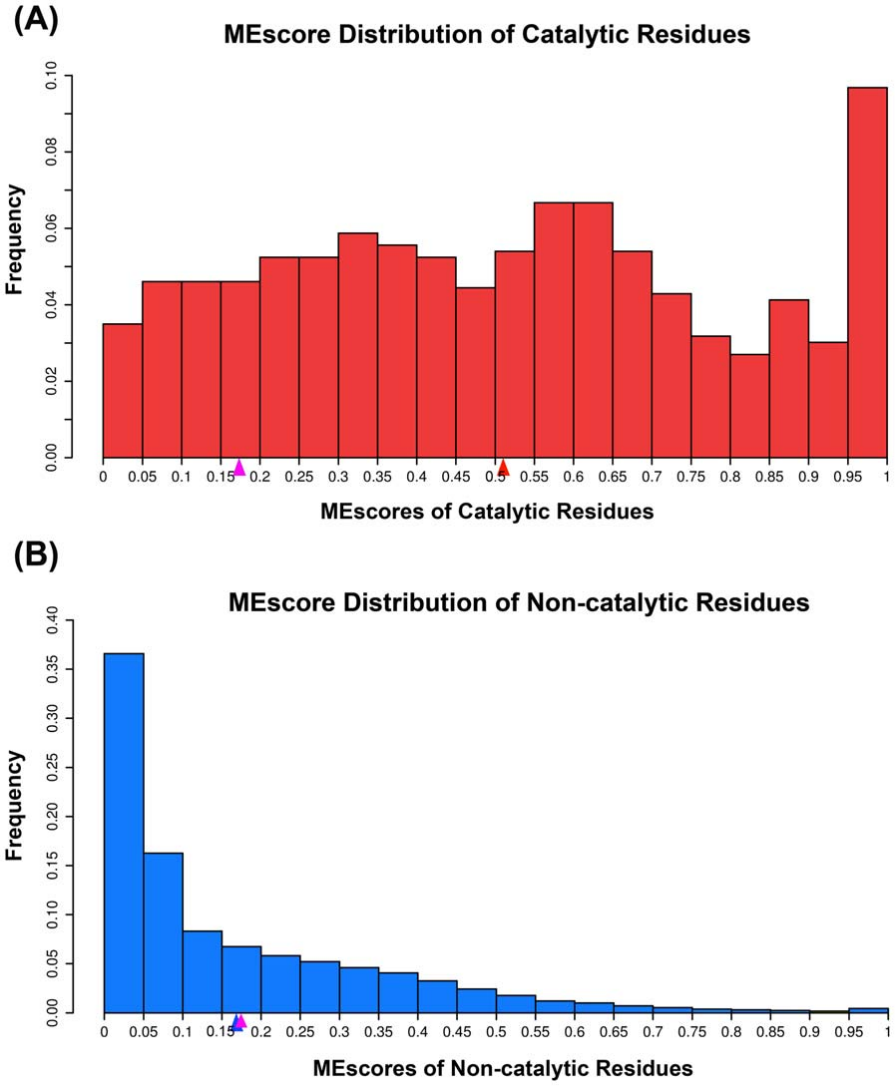
The distribution of MEscores. Panel A and Panel B represent the distributions of MEscore in catalytic and non-catalytic residues, respectively. The pink triangle in *x*-axis represents the average MEscore of all residues in the enzyme dataset, while the red (Panel A) and blue triangle (Panel B) denote the average MEscores of catalytic residues and non-catalytic residues, respectively.

Since the calculation of MEscore requires a training dataset, we applied five-fold cross validation tests to evaluate the performance of MEscores for all the catalytic residues in the enzyme dataset. Briefly, the enzyme dataset was divided into five subsets with roughly equal numbers of protein domains. At each cross-validation step, four subsets were merged into a training dataset to infer 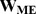 and the MEscores of the residues in the remaining subset (i.e. test dataset) were calculated using the established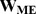 from the training dataset. After completing the five-fold cross validation tests, we obtained the distribution of MEscores for catalytic and non-catalytic residues based on the whole enzyme dataset (Figure 3). The average MEscore for all residues is 0.172. As shown in Figure 3A, a significant portion of catalytic residues (>85%) have MEscore values larger than the average for all residues. In contrast, approximately 35% of the non-catalytic residues have MEscores larger than the average value (Figure 3B). Meanwhile, the average MEscore for the catalytic residues is 0.511, which is in sharp contrast to 0.169 for the non-catalytic residues. Therefore, the MEscores for catalytic residues are significantly higher than non-catalytic residues (Wilcoxon rank-sum test, p-value = 8.07e-197), suggesting that MEscore can serve as a useful feature to discriminate catalytic from non-catalytic residues. Although the five subsets were compiled randomly, they share a reasonably similar distribution of MEscore (Figure S1). This indicates that the MEscore feature is generally robust and should achieve stable performance in each subset.

### Performance of MEscore

**Figure 4:**
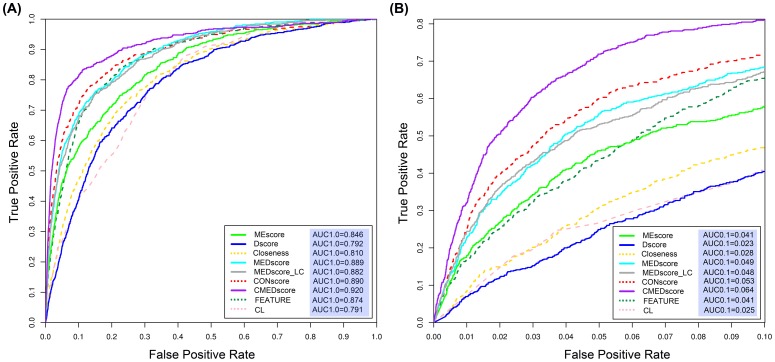
The ROC curves of different features/predictors. Panel A gives the ROC curves at each possible control of false positive rate, while panel B only plots ROC curves at a false positive rate ≤10%.

The performance of MEscore in predicting catalytic residues was evaluated using the receiver operating characteristic (ROC) curves that plot the true positive rate as a function of the false positive rate for all the possible thresholds. Additionally, the prediction performance of MEscore was also quantified by the AUC value (AUC1.0) that represents the corresponding area under the complete ROC curve. In our study, MEscore achieved an AUC1.0 value of 0.846 (Figure 4A). For real-world applications, the ROC curve at a low false positive rate control is more practical. Therefore, the ROC curve at a 10% false positive rate control was plotted and the corresponding AUC value (AUC0.1) was 0.041 (Figure 4B). As listed in Table S2, MEscore does provide a similar performance across five different subsets in the 5-fold cross-validation tests. Note that the above ROC analysis was based on the subset level. That is to say, we generated a ROC curve for each subset and reported the average ROC curve over the generated five ROC curves. We also conducted the ROC analysis on per enzyme basis. Briefly, we generated a ROC curve for each enzyme domain and the resulting ROC curve was averaged over all the 223 domains in the enzyme dataset (Figure S2). Since MEscores were normalized for each enzyme domain, the ROC curves generated in these two different ways are very close (cf. Figure 4 and Figure S2).

To avoid the overestimation of the performance of MEscore, a stringent sequence filter criterion (i.e. the sequence identity between any two sequences should be less than 30%) was applied to compile the enzyme dataset. Considering that a larger dataset may represent more completeness of the known catalytic residues' ME information, we also used a looser sequence filter criterion (i.e. 50% sequence identity cutoff) to obtain an enlarged enzyme dataset containing 765 domains and reassessed the performance of MEscore. Interestingly, there was only a slight increase of the overall performance based on this extended dataset (Figure S3), suggesting that the non-redundant enzyme dataset based on 30% sequence identity has already included sufficient information to deduce MEscore.

Compared to the exposed residues, the buried residues generally have a larger number of neighbors (i.e. having more information about ME). To investigate the performance of MEscore for residues in different structural locations, we classified residues into the buried and the exposed according to their relative solvent accessibility (RSA) values calculated by NACCESS [57]. Then, the ROC curves for the buried and the exposed residues were respectively plotted. As shown in Figure S4, the buried catalytic residues could be more accurately identified compared to the exposed ones. We further investigated the performance of MEscore in different structural folds. As shown in Figure S5, the performance of MEscore varies in different folds.

We further benchmarked MEscore against a simple residue type-based predictor, which was implemented via statistical analysis of the catalytic likelihood (CL) of each residue type. As shown in Figure 4, the performance of MEscore is significantly better than that of CL at varying false positive rate controls. For example, MEscore achieved an increase of 6.3% in terms of AUC1.0 and an increase of 64% in terms of AUC0.1, respectively, compared to CL's performance (AUC1.0 = 0.791 and AUC0.1 = 0.025). The quantitative performance comparison between MEscore and CL indicates that MEscore does capture more valuable information beyond the residue type in predicting catalytic residues.

### Performance comparison between MEscore and Dscore

**Figure 5:**
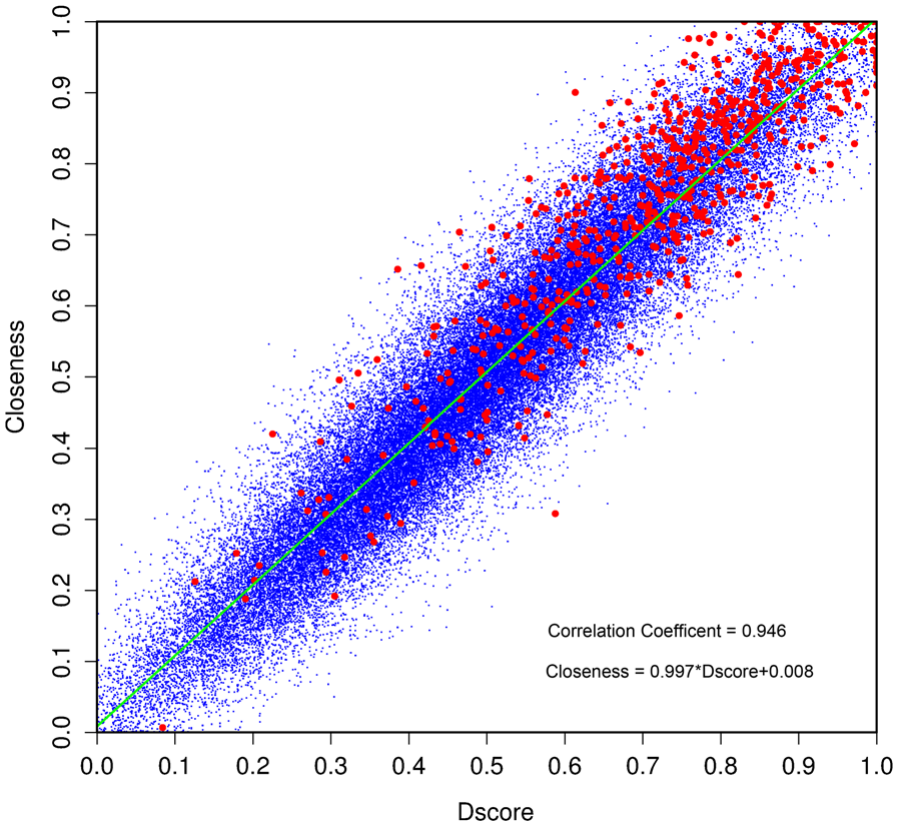
The relationship between Dscore and Closeness. The red points denote catalytic residues, while the blue points represent the non-catalytic residues. The correlation coefficient between Dscore and Closeness is 0.946 as derived by regression equation (i.e. the green line).

Since catalytic residues tend to be located in the center of protein structures, the distance of a residue to the geometrical center of protein has been previously shown to be a powerful structural feature for identifying catalytic residues [24]. In this work, we calculated Dscore for each residue in order to characterize this structural feature and revisited its performance in identifying the catalytic residues based on the benchmark enzyme dataset. In the context of residue interaction networks, the Closeness measure compared favorably with other features in predicting catalytic residues [27], [37]–[39], [43], [47]. Ben-Shimon and Eisenstein found that there was a strong correlation between Dscore and Closeness based on four known enzyme structures [24]. Here, we confirm the presence of this high correlation in the current enzyme dataset [Figure 5; Pearson's correlation coefficient (PCC) = 0.946]. Therefore, Dscore and Closeness do contain similar protein structural information, despite the fact that they are extracted based on different protein structure representations. Since Closeness also describes a residue's geometrical distance to the center of protein structure, it is less likely that a minor conformation change of protein structure will considerably affect the performance of Closeness [39]. Considering that catalytic residues prefer to be located in the center of protein structures, it is more favorable for catalytic residues to cooperate with other neighboring residues during the course of catalysis [54] and have more evolutionary constraints [58]. More importantly, this may be a universal phenomenon for functional residues; for example, other functional important residues also tend to be located in the center of protein structures. Therefore, Closeness has been also used as a feature for predicting single amino acid polymorphisms (SAP) [59] and other functional sites in protein structures [37], [60].

Apparently, MEscore captures different structural information in comparison to Dscore and Closeness; it is necessary to benchmark their performance on the same enzyme dataset. As depicted in Figure 4, at the R_cutoff_ of 9 Å, the AUC1.0 value of MEscore is 0.846 in comparison to 0.810 for Closeness and 0.792 for Dscore. The performance of MEscore is significantly better than Closeness (DeLong's test [61], [62], p-value = 0.000152) and Dscore (DeLong's test, p-value = 2.023e-07). In terms of AUC0.1 (i.e. the area under ≤10% false positive rate control), MEscore achieved an AUC0.1 of 0.041, which is also significantly better than that of Dscore (AUC0.1 = 0.023; Bootstrap test [62], p-value = 1.200e-20) and Closeness (AUC0.1 = 0.028; Bootstrap test, p-value = 2.148e-11).

**Figure 6:**
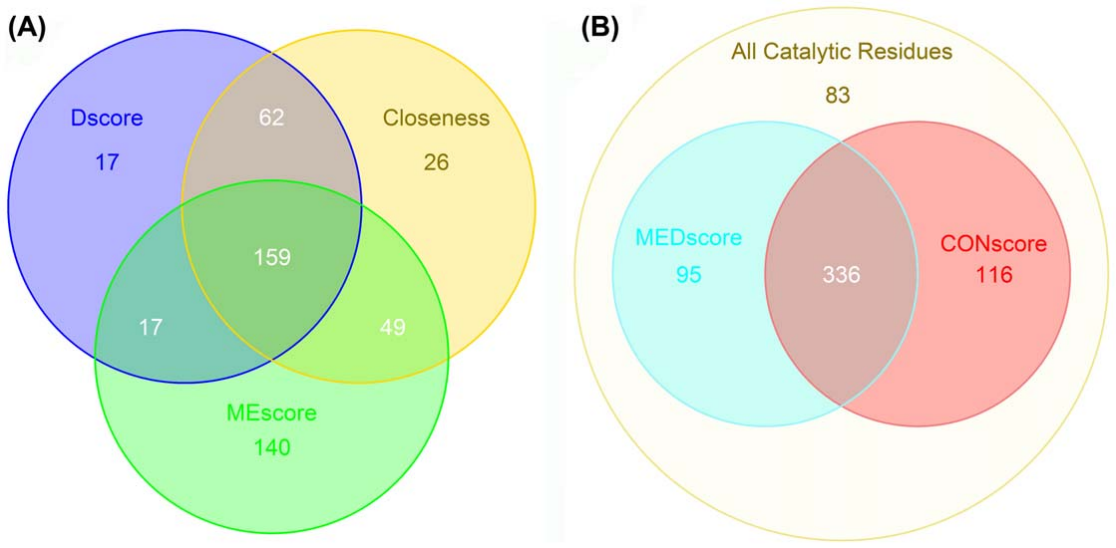
Venn diagrams showing the numbers of catalytic residues identified at a false positive rate ≤10%. Panel A shows the overlapping predictions by three different features (Dscore, Closeness and MEscore), and panel B summarizes the prediction results by MEDscore and CONscore.

In order to analyze the overlapping predictions by MEscore, Dscore and Closeness, we drew a Venn diagram based on their prediction results at the ≤10% false positive rate (Figure 6A). The Venn diagram further confirms that Dscore provided a similar prediction capacity as Closeness. For instance, 221 catalytic residues were consistently predicted by both Dscore and Closeness, accounting for 86.7% of the Dscore and 74.7% of Closeness predictions, respectively. The high number of overlapping predictions indicates again that these two features describe similar structural properties of the protein. On the other hand, only 48.2% and 57.0% of the catalytic residues predicted by MEscore were consistently predicted by Dscore and Closeness, respectively (Figure 6A). Moreover, there is a weak correlation between MEscore and the other two features (PCC = 0.112 for Dscore and PCC = 0.133 for Closeness, respectively). These results suggest that MEscore is strongly complementary to Dscore and Closeness and an integration of MEscore with Dscore or Closeness may result in an even more powerful structural feature. Considering that the mathematical implementation of Dscore is much easier than that of Closeness, only the integration of MEscore and Dscore was carried out in this work.

### Integrating MEscore and Dscore into a novel structural feature

We developed a novel nonlinear integrative structural feature by combining MEscore and Dscore, termed as MEDscore. MEDscore is a modified MEscore with the positional information (i.e. Dscore) taken into account. More details are given in the ‘Materials and Methods’ Section. As illustrated in Figure 4A, MEDscore achieved an AUC1.0 value of 0.889, which is a significantly improved performance than the individual MEscore (DeLong's test, p-value = 3.236e-34) or Dscore (DeLong's test, p-value = 1.219e-34). At the false positive rate control of 10%, the AUC0.1 value of MEDscore is 0.049, which is 113.0% and 19.5% higher than Dscore (Bootstrap test, p-value = 3.385e-60) and MEscore (Bootstrap test, p-value = 5.099e-19), respectively.

As an alternative integration, we also combined MEscore and Dscore in a linear way to form a feature called MEDscore_LC, which is defined as a weighted sum of MEscore and Dscore. MEDscore_LC also achieved a significantly better performance than MEscore and Dscore (Figure 4). For instance, the AUC1.0 value of MEDscore_LC is 0.882, slightly lower than that of MEDscore (DeLong's test, p-value = 0.014). At a false positive rate control of 10%, MEDscore_LC also revealed a slightly lower performance in comparison to MEDscore (Figure 4B). The improved performance of MEDscore and MEDscore_LC further confirms that MEscore and Dscore are complementary with each other. The integrative strategies of MEDscore and MEDscore_LC present several common advantages. For instance, both strategies are simple and easy to implement, and the two new features have clear and interpretable physicochemical meanings. Nevertheless, the integrative strategy of MEDscore is superior to the linear combination used by MEDscore_LC in terms of the prediction performance and whether or not there is a requirement for parameter optimization. Moreover, the construction of MEDscore has offered a novel and important way to integrate more relevant features to improve the prediction performance of catalytic residues as well as other functionally important residues.

### Performance comparison between MEDscore and FEATURE

As a well-established protein functional site predictor, FEATURE extracts a set of features from the structural ME of a query residue and conducts the prediction through a Bayesian classifier [63]. A key idea that MEDscore and FEATURE share is the concept of ME representation. It is of great interest here to benchmark MEDscore against FEATURE. As can be seen from Figure 4A, MEDscore outperformed FEATURE slightly (AUC1.0 = 0.889 vs. 0.874). At the false positive rates of less than 10%, MEDscore was significantly better than FEATURE (AUC0.1 = 0.049 vs. 0.041; DeLong's test, p-value = 2.518e-7) (Figure 4B). Note that another measure MEscore also showed a better performance than FEATURE at the false positive rate control of 5% (0.0146 vs. 0.0135, Figure 4B), yet the AUC1.0 of MEscore was lower than that of FEATURE. However, different to FEATURE that employs high dimensional feature vectors and a machine learning-based algorithm to conduct the prediction, our proposed MEDscore has a clearly defined physicochemical meaning.

### Performance comparison between MEDscore and the residue conservation score CONscore

As a widely used feature, the residue conservation score has proved to be the most effective singular feature in catalytic residue prediction [8]–[12]. In this section, we benchmarked MEDscore against one of the most advanced residue conservation measures, i.e. CONscore. The CONscore was extracted from the Consurf_DB database [64], which used the Rate4Site algorithm [16] to measure the conservation score for each residue in the protein. As shown in Figure 4, MEDscore showed a comparable performance with CONscore (AUC1.0 = 0.890; DeLong's test, p-value = 0.400). However, the performance of MEDscore was slightly lower than that of CONscore at the false positive rate control of ≤10% (AUC0.1 = 0.049 vs. 0.053; Bootstrap test, p-value = 0.018).

In addition we further benchmarked MEDscore against two common conservation scoring methods (i.e. the Shannon entropy [14] and Shannon entropy with residue properties [65]).As shown in Figure S6, the performance of MEDscore is better than these two conservation scores, indicating that MEDscore is competitive with different residue conservation measures.

Since MEDscore and CONscore target and capture different properties in proteins, their inter-correlation should be generally low. Indeed, for all the catalytic residues in the dataset, the PCC between MEDscore and CONscore was only 0.192, suggesting that there may be a large complementarity between the two features. Furthermore, we also generated the Venn diagram based on their prediction results at the ≤10% false positive rate control (Figure 6B). The Venn diagram further suggests that CONscore and MEDscore are complementary with each other to some extent. 53.4% of the catalytic residues that were not identified by CONscore, were correctly predicted by MEDscore. On the other hand, 58.3% of the catalytic residues that were not identified by MEDscore, could be correctly predicted by CONscore (Figure 6B). Therefore, the integration of these two features may lead to a more accurate and comprehensive predictor of catalytic residues.

To examine the feasibility and advantage of combining MEDscore and CONscore, we further integrated CONscore and MEDscore to form a new feature termed as CMEDscore using the weighted sum of CONscore and MEDscore. As shown in Figure 4A, CMEDscore achieved the highest AUC1.0 value of 0.920, outperforming MEDscore and CONscore with an increase in AUC1.0 of 3.48% (DeLong's test, p-value = 6.627e-25) and 3.37% (DeLong's test, p-value = 2.202e-06), respectively. At the false positive rate control of 10%, CMEDscore correctly recognized 81.1% of the catalytic residues and its corresponding AUC0.1 was 0.064. This is also remarkably higher than MEDscore (Bootstrap test, p-value = 2.681e-37) and CONscore (Bootstrap test, p-value = 1.132e-15). Taken together, these results demonstrate that MEDscore has a competitive performance and an excellent complementarity to CONscore.

### Case studies

**Figure 7:**
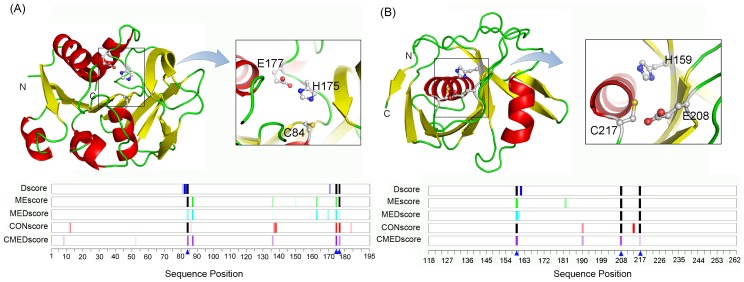
Two case studies illustrating the prediction performance of different features at a false positive rate control of 3%. Panel A shows the predicted catalytic residues of TrpG (the small domain of anthranilate synthase; PDB entry: 1QDL), and panel B gives the predictions of diaminopimelate (DAP) epimerase (PDB entry: 1BWZ). Top parts: Protein structures are represented by cartoon ribbons and the corresponding catalytic residues are highlighted by ball-and-stick-models, as seen in the insets. Lower parts: The blue triangles represent the sequence positions of the catalytic residues. With respect to the prediction results of each feature, the sequence positions of the predicted catalytic residues are marked using colored bars, with a higher score corresponding to a more saturated color. The black bars denote catalytic residues which a corresponding feature failed to predict.

We performed two case studies to illustrate the prediction performance of all the features we have developed. In the first case study, we predicted the catalytic residues of a tryptophan biosynthesis related enzyme. Tryptophan is an important substrate for protein biosynthesis in microorganisms and plants [66]. The first step in synthesizing tryptophan is the biosynthesis of anthranilate from chorismate, catalyzed by anthranilate synthase (AnthS, PDB entry: 1QDL [67]). The small domain of AnthS (TrpG, SCOP family index: c.23.16.1) is a glutamine amidotransferase (EC 4.1.3.27) that hydrolyzes glutamine and transfers the ammonia group to a substrate to form a new carbon-nitrogen group [66], [68]. The function of TrpG is carried out through a catalytic triad of the active site (C84, H175 and E177) [67]. In Figure 7A, the cartoon representation of TrpG and the prediction performance at a false positive rate control of 3% are illustrated. At such a low false positive rate control, Dscore failed to recognize any catalytic residue and MEscore correctly identified only one catalytic residue (H175). Remarkably, MEDscore correctly identified all three catalytic residues, suggesting the robustness of this nonlinear combination between MEscore and Dscore. In fact, MEDscore in this case also performed better than CONscore. We also found that CMEDscore achieved an even better performance, with all three catalytic residues ranked as the top hits according to the CMEDscore values.

The second case study concerns the catalytic residue prediction of diaminopimelate (DAP) epimerase (EC 5.1.1.7), a typical member of pyridoxal phosphate (PLP)-independent amino acid racemase involved in lysine biosynthesis [69]. The structure of DAP epimerase was characterized from *Haemophilus influenzae* (PDB entry: 1BWZ), consisting of two homology domains [70]. In the C-terminal domain (residues 118–262, SCOP family index: d.21.1.1), three catalytic residues (H159, E208 and C217) directly contribute to the catalytic process. Figure 7B presents the performance of different features at the false positive rate control of 3%. At this rigorous control, the performance of both of MEDscore and CONscore was weak. MEDscore only correctly detected one of the three catalytic residues while CONscore identified none. In comparison, CMEDscore was able to correctly predict all three catalytic residues. These results indicate that there indeed exists a complementarity between CONscore and MEDscore.

It is noteworthy that both of the two query proteins (TrpG and DAP epimerase) share less than 40% sequence identity with any other domains in the enzyme dataset used in this work. The findings from the above case studies provide supportive evidence that MEDscore and its integration with CONscore (i.e. CMEDscore) can favourably identify a large portion of catalytic residues from the given protein structure with better accuracy. This suggests that these new features can be efficiently employed for practical applications.

### Conclusion

A number of structural features have been developed to identify catalytic residues from enzyme structures. However, the performance of most features is not comparable to the most powerful sequence-based feature, namely, the residue conservation score. In this work we develop a novel promising structural feature termed as MEDscore for the identification of catalytic residues. The superior performance of MEDscore can be ascribed to its capability of capturing the intrinsic ME and geometrical location properties of the residues. In particular, it allows the ME of a residue to be converted into a series of spatially neighboring residue pairs such that the likelihood of belonging to the catalytic ME could be deduced from a pre-existing enzyme dataset. To the best of our knowledge, this research represents the first endeavor to characterize ME of a residue based on this strategy. From the practical perspective, we find that the proposed MEDscore performs better in catalytic residue prediction when being integrated with other features, such as CONscore. Moreover, it should be noted that MEDscore may be the first structural feature that shows a competitive performance compared to the residue conservation score. We anticipate that this novel structural feature can be applied to reliably identify catalytic residues, facilitate the functional annotation of structural genomics targets and improve our understanding of the complex sequence-structure-function relationships of enzymes.

## Materials and Methods

### Benchmark enzyme dataset

The benchmark enzyme dataset used in this study was extracted from the Catalytic Site Atlas (CSA) database (version 2.2.12) [71]. Total of 7,124 entries with catalytic residues annotated directly in the literature were extracted. These entries were mapped onto the SCOP database (version 1.75) [72] and the corresponding PDB files were downloaded from the ASTRAL database (http://astral.berkeley.edu/pdbstyle-1.75.html) [73]. These enzymes were further filtered based on the following criteria: a) the sequence identity between any two sequences should be less than 30%; b) the sequence length of any enzyme should be larger than 100; c) the PDB structures with 10 consecutive missing residues were excluded; d) only the PDB structures belonging to four SCOP structural classes (i.e. all-α, all-β, α+β and α/β) were included; e) if an enzyme had two or more NMR structure models in our dataset, only the first model was retained; and f) some enzymes were discarded because that the number of homologous sequences of the enzymes was insufficient to permit an accurate calculation of residue conservation scores. Based on the above criteria, 223 enzyme catalytic domains were retained in our final dataset, covering six top levels of the EC classifications. These 223 enzymes cover 112 folds, 139 superfamilies and 185 families in terms of the SCOP classification. In this non-redundant benchmark enzyme dataset, 630 residues are defined as catalytic residues according to the CSA annotation, while the remaining 60,658 residues are regarded as non-catalytic residues. The details about the enzyme dataset are listed in Supporting Information Text S1.

### Definition and Calculation of MEscore

#### The definition of ME in the context of residue interaction networks

Given that a protein structure can be represented as a residue interaction network, residues are viewed as nodes and an edge can be established if the distance between the two residues is less than a distance cutoff (R_cutoff_). The residue interaction network can be represented as an adjacency matrix *D* as follows
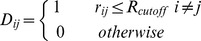
(1)where 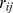 is the spatial distance between residue *i* and *j*. In this work, 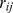 is defined as the shortest distance between any pairs of the heavy atoms (C, N, O, S) in residue *i* and *j*.

In the context of residue interaction networks, the direct neighboring residues constitute the ME of a given residue. In other words, the ME of a residue can be defined using its direct interacting neighbors in the residue interaction network. Here, a 400-dimensional residue pair frequency vector called 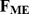 is used to represent the ME information of a residue, which is defined as
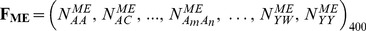
(2)Note that the residue pair representation in 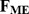 is orientation-dependent. More specifically, the residue pair *A_m_A_n_* has the orientation from the query residue 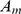 to its spatially neighboring residue 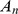. It should be noted that the residue pairs between any two neighboring residues of 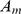 are not taken into account. The value of each feature, such as 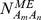, denotes the number of the corresponding residue pair involved in the ME, which can be readily extracted from the pre-computed adjacency matrix *D* of the protein structure. Although 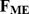 of a residue is defined as a 400-dimensional vector, it is highly sparse in nature and only contains 20 parameters with potential non-zero values.

#### The residue pair weight coefficient vector

Our hypothesis is that spatially neighboring residue pairs of the catalytic residues should have a specific frequency distribution and such specificity can be determined and used to identify catalytic residues in a given protein structure. To explore the frequency distribution, we introduce a residue pair weight coefficient vector for each residue in a protein structure, which is expressed as
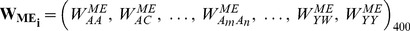
(3)Here 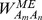 is calculated as
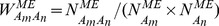
(4)where 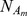 and 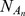 are the numbers of residues 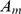 and 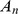 in the ME of the query residue, respectively. Note that the query residue itself is also included when counting 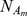.

The overall 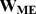 vector for catalytic residues is measured by averaging all the weighted vectors of the related catalytic residues:
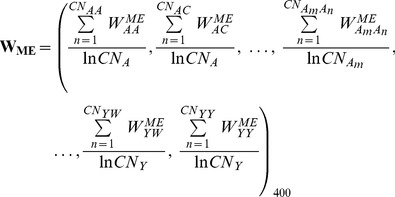
(5)where 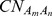 is the total number of residue pairs of 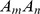 in all the MEs of catalytic residues, while 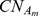 represents the total number of catalytic residues of type 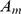.

### MEscore

MEscore is proposed to measure the likelihood of a query residue being catalytic, which is derived using the following equation:
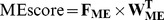
(6)Here 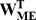 is the transposed matrix of 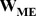. The MEscores of all residues in a protein structure are further normalized using the following equation:
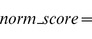
(7)In this study, six different *R_cutoff_* values (from 3 to 13 Å at an interval of 2Å) were examined in order to obtain the optimal one. As a result, MEscore almost achieved the maximal performance when *R_cutoff_* was set as 9 Å (Figure S7). Therefore, the optimal value of *R_cutoff_* was set as 9.0 Å.

### Definition and calculation of Dscore

Since catalytic residues tend to be located in the center of protein structures, we further develop a feature termed as Dscore to characterize this property. Briefly, each residue in a protein structure is represented by its C_α_ atom and the atomic coordinates of the geometrical center of this protein are calculated as follows:
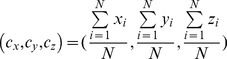
(8)where 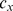, 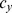 and 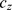 are the coordinates of the center of the protein structure; *x_i_*, *y_i_* and *z_i_* are the trajectory of the C_α_ atom in residue *i*; while *N* is the total number of residues in the protein. The distance between a residue *i* and the center of the structure (i.e. Dscore_i_) is then calculated as
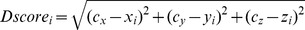
(9)The Dscore values of each protein are normalized using the above Eq. (7). After normalization, its values in a protein vary in the range from 0 to 1. To transform Dscore from a dissimilarity to similarity measure, each Dscore is subtracted by 1. Thus, a residue near the center of the protein structure presumably has a relatively high Dscore.

### Definition and calculation of MEDscore and MEDscore_LC

Dscore is a global feature that describes the positional information of a residue in protein, while MEscore is a local feature that describes the local environment surrounding the residue of interest. As these two types of features capture different and complementary information of the catalytic residue, we integrate them to constitute a novel feature in a nonlinear manner. The rationale behind this nonlinear integration is to construct a refined MEscore, termed as MEDscore, where the positional information (Dscore) of each residue involved in the ME representation is also considered. To achieve this, 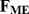 is firstly modified to 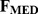:
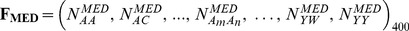
(10)where 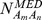 is calculated as
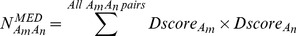
(11)where 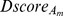 and 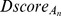 are the Dscore of the query residue 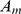 and its neighboring residue 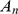, respectively. Note that the definition of 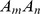 is the same as described in 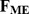. For each occurring residue pair 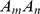, the two corresponding Dscore values are multiplied to obtain a coefficient. 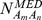
corresponds to the summation of the corresponding coefficients for all the observed 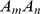. Then, the residue pair weight coefficient vector for each residue in a protein structure is modified to
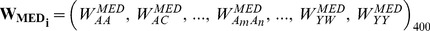
(12)Here 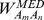 is calculated as
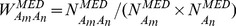
(13)where 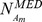 stands for the summation of the Dscores for all the occurring 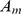 in the ME and 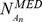 represents the summation of the Dscores for all the occurring 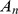 in the ME, respectively. Similar to Eq.(4), the query residue itself is also considered when counting 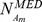.

The overall weighted vector (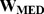) for catalytic residues is measured by averaging all the weighted vectors of the related catalytic residues:
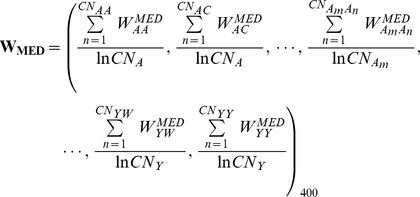
(14)In the above equation, 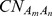 and 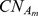 have the same meanings as in Eq. (5).

Finally, a nonlinear combination between MEscore and Dscore is obtained using the following equation:
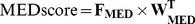
(15)To ensure that the MEDscores of all residues in a protein structure range from 0 to 1, the original MEDscores are further normalized by Eq. (7).

As an alternative combination, we linearly combine MEscore and Dscore into another feature called 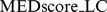, which is defined as
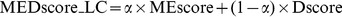
(16)To determine the optimal value of *α*, we benchmarked the performance of MEDscore_LC using different *α* values, ranging from 0.0 to 1.0 at an interval of 0.05. The optimal *α* corresponded to the maximal AUC1.0. In this work, the optimal value of *α* was assigned to 0.55.

### Other existing features and predictors

#### Catalytic likelihood of each residue (CL)

The CL value of each residue can be inferred from the benchmark enzyme dataset, defined as
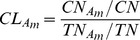
(17)where 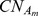 denotes the number of residue 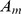 as catalytic, 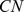 is the total number of catalytic residues, 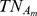 is the number of residue 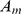, and 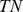 is the total number of residues, respectively. For a given residue, the corresponding value of CL can be used to predict whether a residue is catalytic or not.

#### Closeness

Derived from residue interaction networks, Closeness has been previously shown to be a powerful feature in predicting catalytic residues [27], [37], [39], [43], [47]. In this study, Closeness of a given residue *i* in the residue interaction network of a protein is calculated as
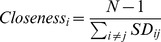
(18)where *N* is the number of residues in the protein structure and *SD_ij_* is the shortest path between residues *i* and *j*. To construct the residue interaction network, the value of *R_cutoff_* was set as 9.0 Å. Note that the Closeness values also need to be further normalized using Eq. (7).

#### FEATURE

FEATURE employs a Bayesian classifier to predict protein functional sites [63]. The input feature vector of FEATURE is constructed from the ME surrounding the query residue, including atom properties, residue properties, partial charge, solvent accessibility etc. We downloaded the stand-alone version of FEATURE (version 3.0) from https://simtk.org/home/feature, and evaluated its performance based on the benchmark enzyme dataset in this study. For performance comparison, as reported in [46], the ratio of catalytic to non-catalytic residues in the training stage was set as 1∶6.

#### Residue conservation score

The residue conservation scores of the enzymes were directly obtained from the ConSurf-DB database [64], which consists of pre-calculated conservation scores for each protein structure. In order to detect functionally important residues in protein structures, ConSurf-DB employs the Rate4Site algorithm [16] to compute the conservation score of each residue based on the generated MSA. The criteria to generate the MSA are detailed in [64]. The number of sequences in the MSAs of the 223 enzyme domains ranges from 8 to 199 and the average number is 124. Approximately 90% of the enzymes have more than 50 aligned sequences in their respective MSAs, while only two enzymes have less than 10 aligned sequences in the corresponding MSAs. In addition to the use of an empirical Bayesian inference, Rate4Site also takes into account the phylogenic relations within proteins. Likewise, the pre-calculated conservation score (CONscore) of each residue is further normalized using Eq. (7).

### Linear combination of CONscore and MEDscore

Similar to the construction of 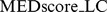, we further integrate CONscore and MEDscore into a new feature, defined as CMEDscore:
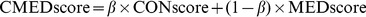
(19)Similar to the determination of the optimal α in Eq. (16), the value of *β* was optimized to attain the maximal AUC1.0 of CMEDscore. In the current enzyme dataset, the optimal value of *β* was set as 0.70.

### Performance assessment of different features or predictors

In this work, we used five-fold cross validation tests to evaluate the performance of the six features/predictors, i.e. MEscore, MEDscore, MEDscore_LC, CL, FEATURE and CMEDscore. In particular, the benchmark enzyme dataset was randomly divided into five subsets and each subset contained roughly equal number of protein domains (the SCOP entries of these five subsets are available in Text S1). In each cross-validation evaluation step, four subsets were merged into a training dataset to infer the residue pair weight vectors (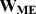 and 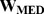) and the remaining were utilized as a test dataset to assess the performance of each feature. The final performance of this feature was averaged over all of the five subsets. We also randomly repeated the subset partition three times and similar results were obtained for each feature/predictor. Since Dscore, Closeness and CONscore do not require a training phase, the performance of these three features was evaluated directly on the whole dataset rather than the five-fold cross validation tests.

We used the ROC curve and two corresponding parameters (AUC1.0 and AUC0.1) to assess the overall performance of each feature. In this work, the ROC curve was prepared on per subset basis. Briefly, we generated a ROC curve in each subset and the overall ROC curve on the whole dataset was averaged over the generated five ROC curves. Note that the ROC curve can also be generated on per enzyme basis. That is to say, we can plot a ROC curve in each enzyme domain and obtain the average ROC curve on the 223 enzyme domains. Since normalization at the domain level was conducted for each feature, the ROC curves based on the above two strategies should generate close results. For comparison, the ROC curves on per enzyme basis are also shown in Figure S2.

### Analysis and Visualization

All computational and analytic scripts were written in Perl/R languages. The implemented R packages included ROCR [74] and pROC [62] for ROC analysis and visualization, as well as igraph [75] for network parameter calculation. All the figures were prepared using either R (http://www.r-project.org/) or PyMol (http://www.pymol.org/).

## Supporting Information

Figure S1 **The MEscore distribution across the five subsets in 5-fold cross-validation tests.**
(PDF)

Figure S2 **The ROC curves of different features/predictors on per enzyme basis.**
(PDF)

Figure S3 **The ROC curves of MEscore and MEDscore based on two different datasets.**
(PDF)

Figure S4 **Performance of MEscore in predicting the buried and the exposed catalytic residues.**
(PDF)

Figure S5 **The performance variation of different features/predictors in different structural folds.**
(PDF)

Figure S6 **The ROC curves of MEDscore and two simple conservation scoring methods (i.e. the Shannon entropy and Shannon entropy with residue properties).**
(PDF)

Figure S7 **The performance of MEscore and MEDscore at different** ***R_cutoff_*** **values.**
(PDF)

Text S1 **This file contains the FASTA sequences and the experimentally verified catalytic residues of 223 enzymes used in this work.** The SCOP entries of the five subsets used in the five-fold cross validation tests are also given.
(DAT)

Table S1
**This file contains the calculated weight coefficient vector** 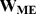 **, which is represented by a 20×20 amino acid matrix.** Note that the listed 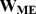 was derived from the whole enzyme dataset with 223 enzymes.(DOC)

Table S2 **The performance of MEscore in five subsets.**
(DOC)
