# Supplementary material for: Identification of Catalytic Residues Using a Novel Feature that Integrates the Microenvironment and Geometrical Location Properties of Residues
Source: PLoS One. 2012 Jul 19;7(7):e41370. doi: 10.1371/journal.pone.0041370 (PMC3400608; doi:10.1371/journal.pone.0041370)
Supplement: Figure S1 — The MEscore distribution across the five subsets in 5-fold cross-validation tests. (PDF) [file pone.0041370.s001.pdf]

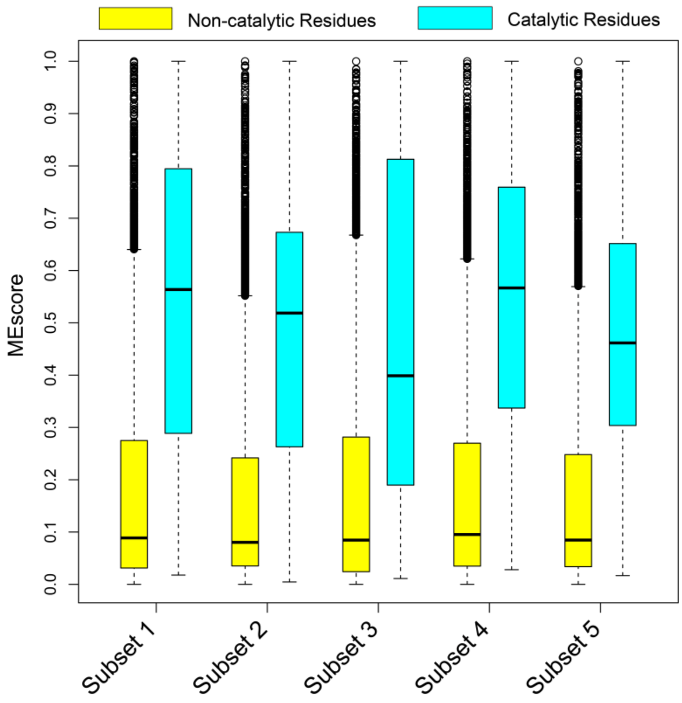

Figure S1. The MEscore distribution across the five subsets in 5-fold cross-validation tests. Generally, MEscores have similar distributions in different subsets. Compared with other subsets, the catalytic residues in subset 3 have lower MEscores, possibly due to the presence of more unique folds in this subset.
