# Supplementary material for: Identification of Catalytic Residues Using a Novel Feature that Integrates the Microenvironment and Geometrical Location Properties of Residues
Source: PLoS One. 2012 Jul 19;7(7):e41370. doi: 10.1371/journal.pone.0041370 (PMC3400608; doi:10.1371/journal.pone.0041370)
Supplement: Figure S2 — The ROC curves of different features/predictors on per enzyme basis. (PDF) [file pone.0041370.s002.pdf]

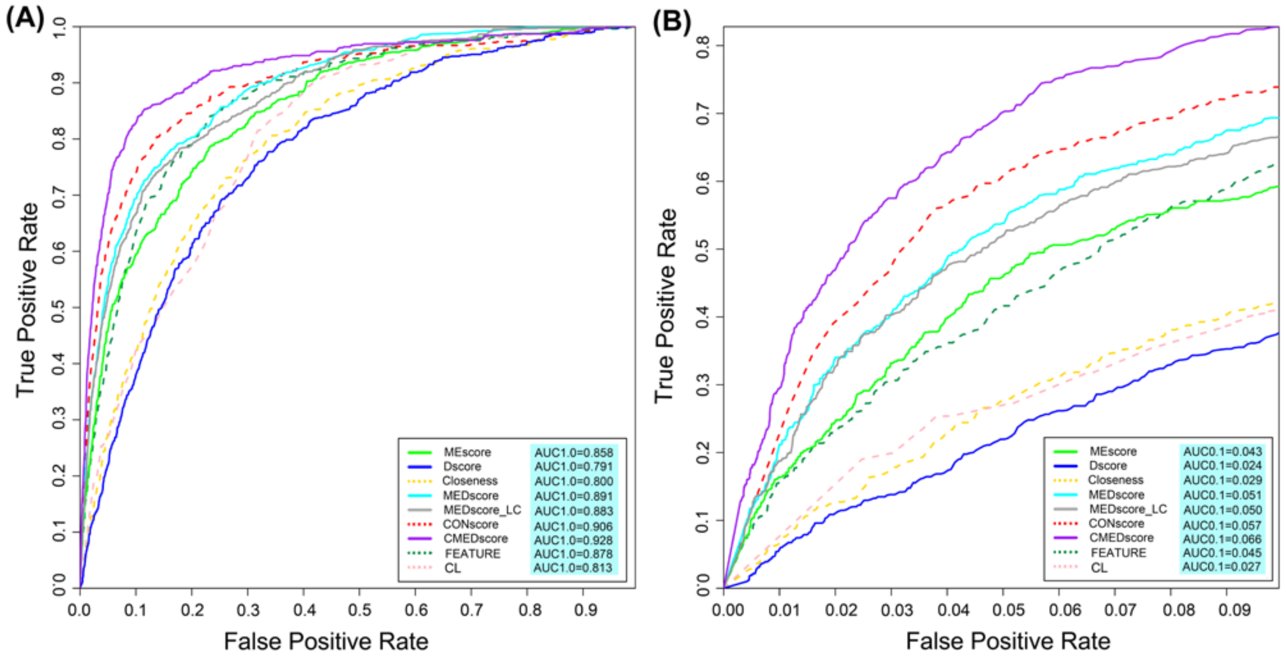

Figure S2. The ROC curves of different features/predictors on per enzyme basis. A ROC curve was prepared for each enzyme domain and the reported ROC curve was averaged over the 223 enzyme domains. Panel A gives the ROC curves at each possible false positive rate control, while panel B only plots ROC curves at a false positive rate ≤ 10%.
