# Supplementary material for: Identification of Catalytic Residues Using a Novel Feature that Integrates the Microenvironment and Geometrical Location Properties of Residues
Source: PLoS One. 2012 Jul 19;7(7):e41370. doi: 10.1371/journal.pone.0041370 (PMC3400608; doi:10.1371/journal.pone.0041370)
Supplement: Figure S3 — The ROC curves of MEscore and MEDscore based on two different datasets. (PDF) [file pone.0041370.s003.pdf]

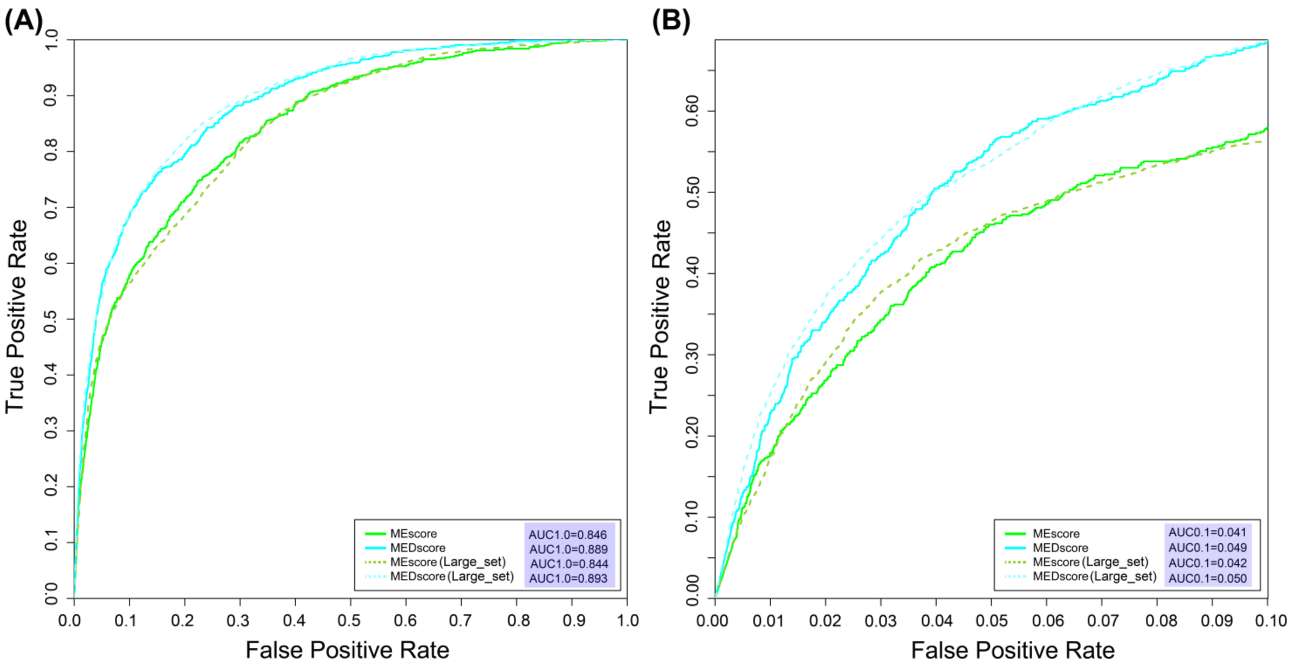

Figure S3. The ROC curves of MEscore and MEDscore based on two different datasets. The ROC curves with solid lines are based on the 223 enzyme domains, while the dashed ROC curves are based on a larger enzyme dataset, in which the identity of any two sequences is less than 50%. The large enzyme set (i.e. Large_set) contains 765 domains, including 2,114 catalytic residues and 205,000 non-catalytic ones. Panel A gives the ROC curves at each possible false positive rate control, while panel B only plots ROC curves at a false positive rate ≤ 10%.
