# Supplementary material for: Identification of Catalytic Residues Using a Novel Feature that Integrates the Microenvironment and Geometrical Location Properties of Residues
Source: PLoS One. 2012 Jul 19;7(7):e41370. doi: 10.1371/journal.pone.0041370 (PMC3400608; doi:10.1371/journal.pone.0041370)
Supplement: Figure S4 — Performance of MEscore in predicting the buried and the exposed catalytic residues. (PDF) [file pone.0041370.s004.pdf]

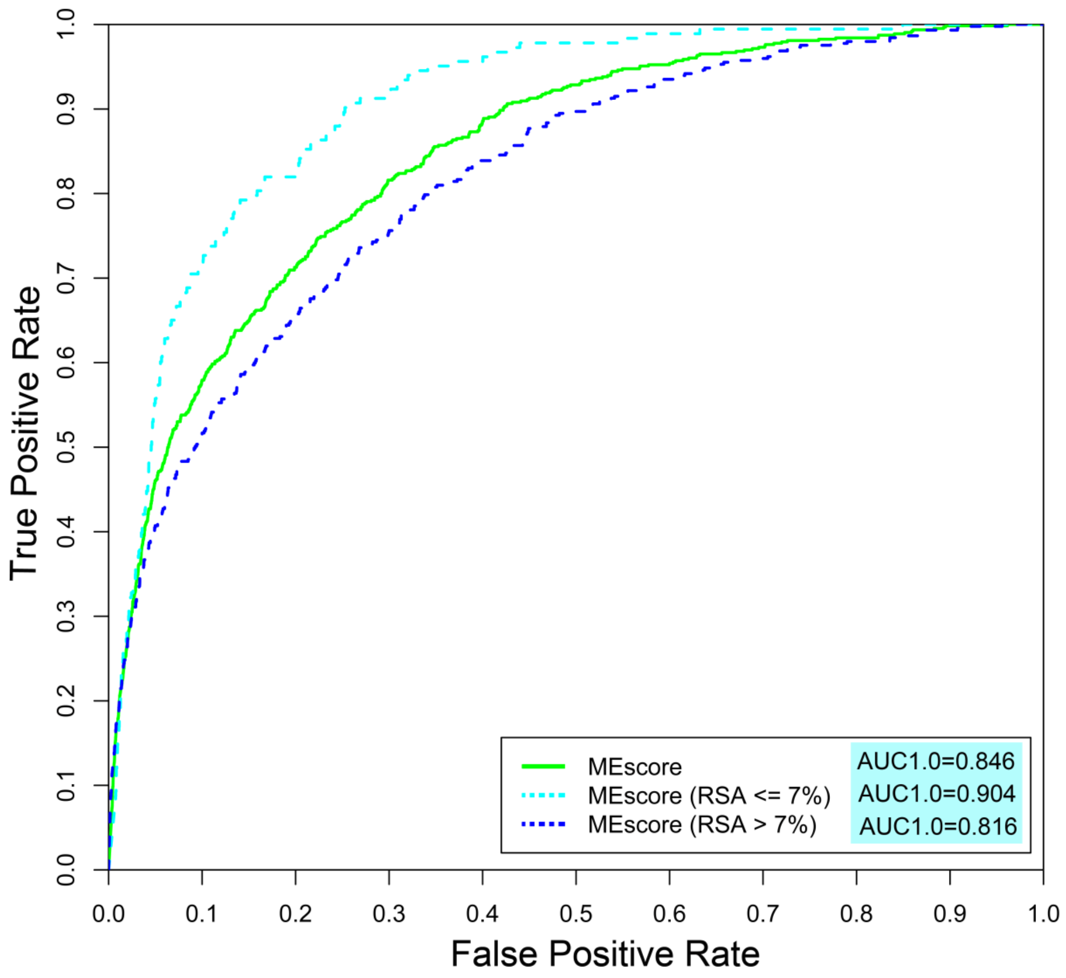

Figure S4. Performance of MEscore in predicting the buried and the exposed catalytic residues. Residues were classified into the buried and the exposed according to their relative solvent accessibility (RSA) values calculated by NACCESS. The buried and exposed residues correspond to the RSA values ≤7% and >7%, respectively. In addition to the average ROC curve based on the whole dataset, two ROC curves concerning only buried residues and exposed residues were respectively plotted.
