# Supplementary material for: Identification of Catalytic Residues Using a Novel Feature that Integrates the Microenvironment and Geometrical Location Properties of Residues
Source: PLoS One. 2012 Jul 19;7(7):e41370. doi: 10.1371/journal.pone.0041370 (PMC3400608; doi:10.1371/journal.pone.0041370)
Supplement: Figure S5 — The performance variation of different features/predictors in different structural folds. (PDF) [file pone.0041370.s005.pdf]

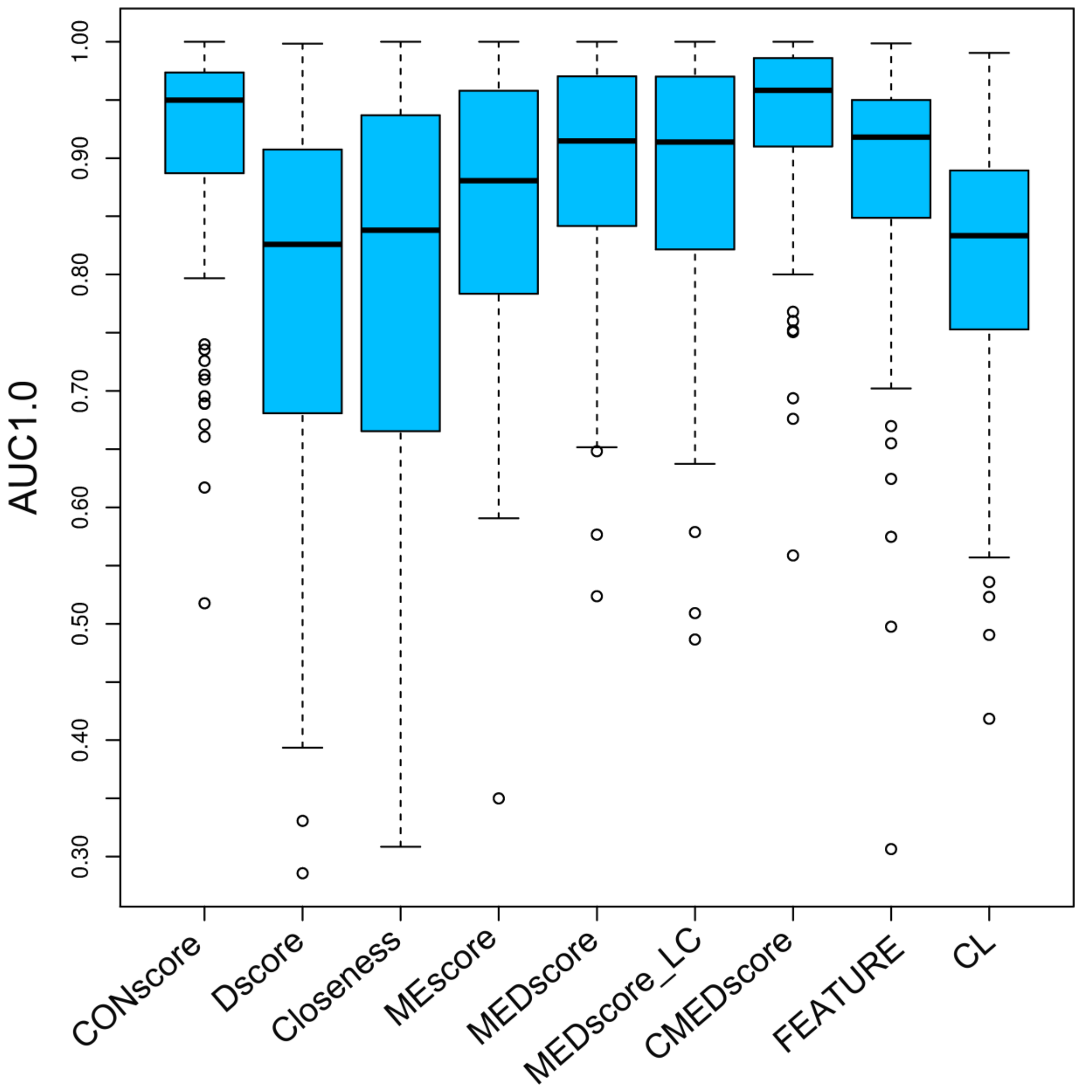

Figure S5. The performance variation of different features/predictors in different structural folds. Generally, all the features/predictors show a variable performance in different folds. Comparatively, the geometrical properties-related features (e.g. Dscore and Closeness) are more variable.
