# Supplementary material for: Identification of Catalytic Residues Using a Novel Feature that Integrates the Microenvironment and Geometrical Location Properties of Residues
Source: PLoS One. 2012 Jul 19;7(7):e41370. doi: 10.1371/journal.pone.0041370 (PMC3400608; doi:10.1371/journal.pone.0041370)
Supplement: Figure S6 — The ROC curves of MEDscore and two simple conservation scoring methods (i.e. the Shannon entropy and Shannon entropy with residue properties). (PDF) [file pone.0041370.s006.pdf]

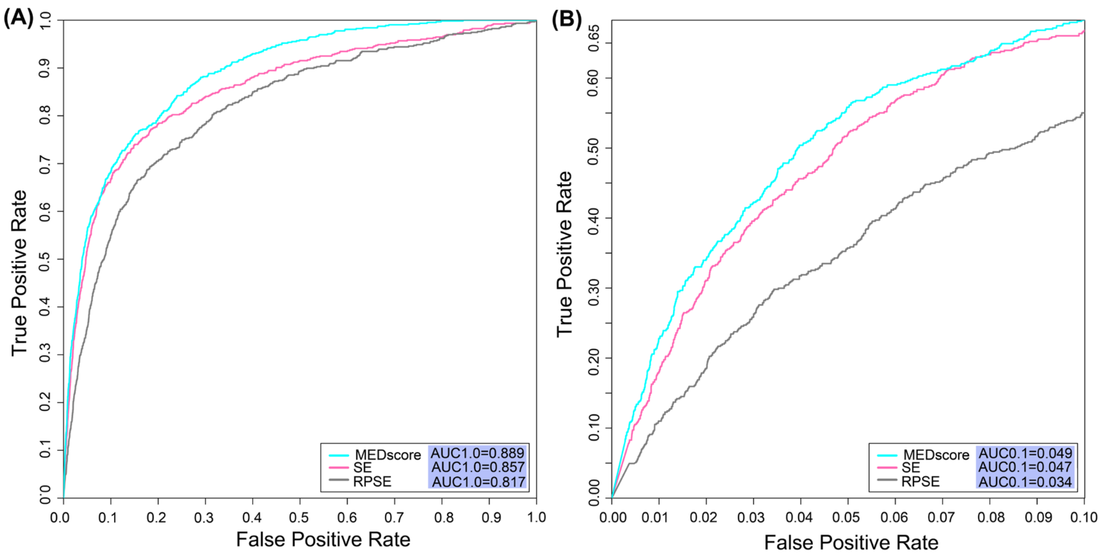

Figure S6. The ROC curves of MEDscore and two simple conservation scoring methods [i.e. the Shannon entropy (SE) and Shannon entropy with residue properties (RPSE)]. SE of a residue is defined as: $SE_i = -\sum_{\chi \in AA} p_i(\chi) \times \ln p_i(\chi)$ where χ belongs to one of the 20 amino acids (AA) and $p_i(\chi)$ is the probability of χ in the ith column of the multiple sequence alignment (MSA). To obtain the RPSE value of a given residue, we first classified 20 amino acids into the following groups: aliphatic [AVLIMC], aromatic [FWYH], polar [STNQ], positive [KR], negative [DE] and [GP]. Then, the RPSE value could be obtained by computing the entropy according to this classification. The same MSAs used in the calculation of CONscore were used and the program developed by Capra and Singh (2007) was employed to determine SE and RPSE.
