# Supplementary material for: Identification of Catalytic Residues Using a Novel Feature that Integrates the Microenvironment and Geometrical Location Properties of Residues
Source: PLoS One. 2012 Jul 19;7(7):e41370. doi: 10.1371/journal.pone.0041370 (PMC3400608; doi:10.1371/journal.pone.0041370)
Supplement: Figure S7 — The performance of MEscore and MEDscore at different Rcutoff values. (PDF) [file pone.0041370.s007.pdf]

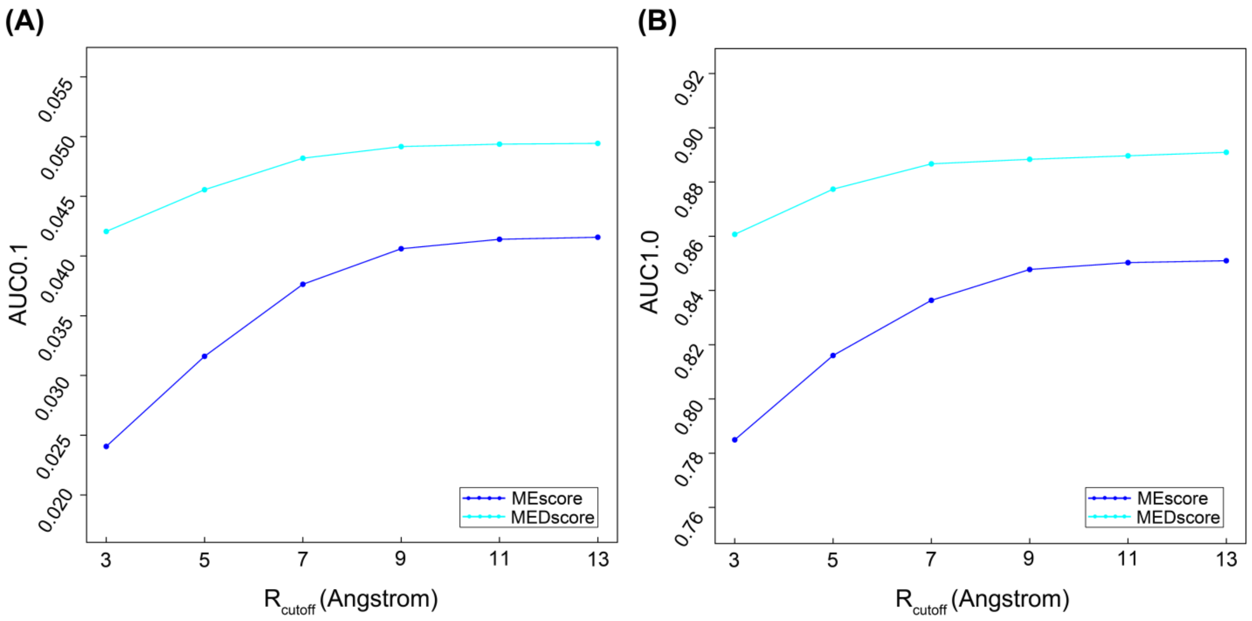


**Figure S7.** The performance of MEscore and MEDscore at different $R_{cutoff}$ values. This shows that a choice of $R_{cutoff}$=9 Å is justified. Note that the performance remains constant using larger $R_{cutoff}$ values (e.g. $R_{cutoff}$ = 11 and 13 Å). Generally, a $R_{cutoff}$ of 13Å should result in a quite large size of ME, in which some residues not related to catalytic process may be taken into account. However, it seems that the performance of MEscore and MEDscore is not affected by the potential noise caused by the increased ME size, which will leave an open question for further investigation.
