# Supplementary material for: Identification of Catalytic Residues Using a Novel Feature that Integrates the Microenvironment and Geometrical Location Properties of Residues
Source: PLoS One. 2012 Jul 19;7(7):e41370. doi: 10.1371/journal.pone.0041370 (PMC3400608; doi:10.1371/journal.pone.0041370)
Supplement: Table S1 — This file contains the calculated weight coefficient vector , which is represented by a 20×20 amino acid matrix. Note that the listed was derived from the whole enzyme dataset with 223 enzymes. (DOC) [file pone.0041370.s009.doc]

**Table S1. The calculated weight coefficient vector**$\mathbf{W}_{\mathbf{ME}}$

Represented by a 20×20 amino acid matrix, the listed $\mathbf{W}_{\mathbf{ME}}$was derived from the whole enzyme dataset (i.e. the 223 enzymes used in this work).

|  | | **Spatially Neighboring Residue** | | | | | | | | | | | | | | | | | | | |
| --- | --- | --- | --- | --- | --- | --- | --- | --- | --- | --- | --- | --- | --- | --- | --- | --- | --- | --- | --- | --- | --- |
|  |  | **A** | **C** | **D** | **E** | **F** | **G** | **H** | **I** | **K** | **L** | **M** | **N** | **P** | **Q** | **R** | **S** | **T** | **V** | **W** | **Y** |
| **Catalytic Residue** | **A** | 0.23 | 0.21 | 0.52 | 0.41 | 0.25 | 0.52 | 0.37 | 0.52 | 0.21 | 0.52 | 0.23 | 0.21 | 0.25 | 0.20 | 0.47 | 0.21 | 0.43 | 0.41 | 0.37 | 0.44 |
|  | **C** | 3.45 | 1.04 | 3.24 | 2.76 | 2.92 | 3.31 | 3.01 | 2.84 | 2.04 | 3.50 | 2.23 | 2.34 | 3.28 | 2.38 | 2.62 | 3.09 | 3.23 | 3.45 | 1.41 | 3.14 |
|  | **D** | 5.47 | 2.77 | 2.26 | 4.27 | 5.38 | 5.89 | 4.53 | 5.20 | 3.13 | 5.39 | 3.96 | 4.46 | 4.33 | 3.58 | 4.55 | 4.94 | 4.76 | 5.13 | 3.52 | 5.12 |
|  | **E** | 4.04 | 2.24 | 3.35 | 1.25 | 4.00 | 3.92 | 3.34 | 4.10 | 2.04 | 3.99 | 3.30 | 3.37 | 3.26 | 3.30 | 3.59 | 3.93 | 3.70 | 4.02 | 2.67 | 3.72 |
|  | **F** | 1.33 | 0.48 | 1.15 | 1.12 | 0.39 | 1.02 | 1.12 | 1.03 | 1.03 | 1.20 | 0.72 | 1.21 | 1.16 | 0.98 | 1.24 | 1.23 | 1.21 | 1.24 | 0.34 | 0.92 |
|  | **G** | 0.88 | 0.53 | 0.82 | 0.41 | 0.79 | 0.49 | 0.71 | 0.82 | 0.44 | 0.79 | 0.61 | 0.54 | 0.69 | 0.45 | 0.52 | 0.82 | 0.70 | 0.86 | 0.32 | 0.65 |
|  | **H** | 6.36 | 2.57 | 6.31 | 5.44 | 5.77 | 6.96 | 2.18 | 5.65 | 4.37 | 6.72 | 4.54 | 5.45 | 5.41 | 4.50 | 5.88 | 6.59 | 6.31 | 6.12 | 4.19 | 5.77 |
|  | **I** | 0.22 | 0.11 | 0.22 | 0.11 | 0.11 | 0.22 | 0.11 | 0.14 | 0.22 | 0.22 | 0.22 | 0.22 | 0.11 | 0.22 | 0.22 | 0.11 | 0.22 | 0.22 | 0.00 | 0.22 |
|  | **K** | 4.12 | 1.48 | 3.90 | 3.53 | 3.79 | 4.26 | 3.00 | 3.76 | 1.28 | 4.09 | 2.62 | 4.02 | 3.50 | 2.33 | 3.31 | 4.06 | 4.04 | 3.99 | 1.72 | 3.33 |
|  | **L** | 0.04 | 0.05 | 0.09 | 0.09 | 0.09 | 0.09 | 0.05 | 0.09 | 0.00 | 0.08 | 0.00 | 0.04 | 0.09 | 0.05 | 0.09 | 0.09 | 0.09 | 0.09 | 0.09 | 0.09 |
|  | **M** | 0.25 | 0.12 | 0.33 | 0.20 | 0.45 | 0.45 | 0.37 | 0.26 | 0.20 | 0.37 | 0.27 | 0.33 | 0.39 | 0.37 | 0.31 | 0.45 | 0.37 | 0.45 | 0.14 | 0.25 |
|  | **N** | 1.72 | 0.84 | 1.68 | 1.53 | 1.74 | 1.84 | 1.51 | 1.62 | 1.13 | 1.90 | 1.25 | 0.76 | 1.59 | 1.26 | 1.59 | 1.66 | 1.66 | 1.79 | 1.08 | 1.51 |
|  | **P** | 0.32 | 0.00 | 0.16 | 0.32 | 0.32 | 0.32 | 0.32 | 0.32 | 0.16 | 0.32 | 0.16 | 0.32 | 0.16 | 0.16 | 0.32 | 0.32 | 0.32 | 0.32 | 0.16 | 0.16 |
|  | **Q** | 1.07 | 0.24 | 0.93 | 0.78 | 0.88 | 1.07 | 0.97 | 0.83 | 0.88 | 0.83 | 0.54 | 0.81 | 0.77 | 0.32 | 0.88 | 0.88 | 1.02 | 1.07 | 0.97 | 0.88 |
|  | **R** | 3.52 | 1.59 | 3.54 | 2.98 | 3.47 | 3.64 | 3.06 | 3.39 | 2.80 | 3.60 | 2.39 | 3.36 | 3.20 | 2.69 | 1.50 | 3.47 | 3.46 | 3.54 | 2.01 | 3.33 |
|  | **S** | 2.08 | 0.75 | 1.75 | 1.74 | 1.90 | 1.94 | 1.65 | 2.04 | 0.87 | 2.02 | 1.18 | 1.81 | 1.64 | 1.29 | 1.74 | 0.98 | 2.05 | 2.06 | 0.92 | 1.55 |
|  | **T** | 1.00 | 0.55 | 0.59 | 0.68 | 0.47 | 1.00 | 0.81 | 0.86 | 0.68 | 0.77 | 0.46 | 0.49 | 0.54 | 0.35 | 0.83 | 0.82 | 0.40 | 0.86 | 0.18 | 0.67 |
|  | **V** | 0.36 | 0.50 | 0.50 | 0.09 | 0.50 | 0.50 | 0.50 | 0.41 | 0.36 | 0.50 | 0.15 | 0.00 | 0.24 | 0.24 | 0.27 | 0.36 | 0.41 | 0.13 | 0.50 | 0.50 |
|  | **W** | 1.09 | 1.07 | 1.31 | 1.13 | 1.05 | 1.18 | 0.90 | 1.00 | 0.95 | 1.22 | 0.74 | 1.27 | 1.03 | 1.09 | 0.99 | 1.18 | 0.79 | 1.13 | 0.39 | 1.09 |
|  | **Y** | 2.20 | 1.17 | 2.46 | 2.63 | 2.37 | 2.31 | 2.13 | 2.30 | 1.68 | 2.65 | 1.60 | 2.11 | 2.29 | 2.14 | 2.60 | 2.60 | 2.53 | 2.07 | 1.84 | 0.88 |
