# Supplementary material for: Identification of Catalytic Residues Using a Novel Feature that Integrates the Microenvironment and Geometrical Location Properties of Residues
Source: PLoS One. 2012 Jul 19;7(7):e41370. doi: 10.1371/journal.pone.0041370 (PMC3400608; doi:10.1371/journal.pone.0041370)
Supplement: Table S2 — The performance of MEscore in five subsets. (DOC) [file pone.0041370.s010.doc]

**Table S2.** The performance of MEscore in each subset in the 5-fold cross-validation tests.

|  | Subset 1 | Subset 2 | Subset 3 | Subset 4 | Subset 5 |
| --- | --- | --- | --- | --- | --- |
| AUC1.0 | 0.852 | 0.849 | 0.817 | 0.858 | 0.856 |
| AUC0.1 | 0.042 | 0.044 | 0.038 | 0.044 | 0.038 |
